# Is science to be trusted? How environmentally active youths relate to science in social media

**DOI:** 10.1177/09636625241249915

**Published:** 2024-05-28

**Authors:** Karin M. Gustafsson

**Affiliations:** Örebro University, Sweden

**Keywords:** discourse analysis, Fältbiologerna, Fridays for Future, science, social media, trust

## Abstract

Research has shown a great distrust among youths toward political representatives, who they demand should “listen to the science.” However, less research has been done on youths’ own trust in science. This study explores and explains how youths who are environmentally active in two different environmental youth organizations relate to science in social media, whether they trust science, and how youths’ relation to science creates a discursive context in which they may develop their identity. The study uses the approach of discourse analysis to examine social media content published on Facebook by *Fridays for Future Sweden* and *Fältbiologerna* (the Swedish Field Biologists). The study shows (i) how subject positions for *scientists* and *youth* are created in relation to one another based on different expressions of youths’ trust in science and (ii) how environmental youth organizations, by identifying with science, make youths important actors in the discourse on climate change.

## 1. Introduction

Previous research on youth in environmental youth organizations such as *Fridays for Future* (FFF) has shown a strong distrust toward political representatives’ capacity and willingness to address the current climate crisis (e.g., Whalström et al., 2020). However, there has been less research on youths’ demand that follows from this distrust that politicians should “listen to the science” (Solli and Mäkitalo, 2022), and there is limited knowledge on environmentally active youths’ relation to science in general. Just a few studies have thus far explored this relation and the deeper meaning of the phrase “listen to the science.” Evensen (2019) has, for example, problematized the rhetorical position of demanding that politics should “listen to the science” and argues that it risks making other important knowledge forms as well as the norms and values of the climate crisis invisible. Kern and Opitz (2021) have shown how countries’ general scientific discourse creates different discursive opportunities for movements such as FFF to have their message resonate and impact climate politics. Finally, Rödder and Pavenstädt (2023) examined the conceptual role of “the science” in the environmental youth movement’s narrative, as well as the roles ascribed to science at the science–policy interface. Similarly, when looking at previous research for studies of environmental nongovernmental organizations that are open to all ages and their relation to science, there are some studies to be found, but they are few. Examples include Eden’s studies on how NGOs engage in boundary work in relation to science, that is, how environmental NGOs negotiate and strengthen their credibility by balancing when they work with scientific knowledge systems and when they work with other knowledge forms (Eden, 2010; Eden et al., 2006). However, no study has yet explored and determined how environmentally active youths’ understanding of science also creates discursive opportunities for them to act in the climate change discourse and how trust in science is of importance in these processes. By exploring environmentally active youths’ relation to science in social media, this study will contribute to filling this knowledge gap with one piece of the puzzle: the discourse on climate change.

Similar to Rödder and Pavenstädt (2023), this study adopts a discourse analytical perspective to explore and explain how environmentally active youths relate to science in social media, whether they trust science, and how youths’ relation to science creates a discursive context in which they develop their identity. This discourse analytical perspective means that text is viewed as part of social practices; the text’s “descriptions are not just *about* something, but they are also *doing* something; that is, they are not merely *representing* some facet of the world, they are also *involved* in that world in some practical way” (Potter, 1996: 47). Thus, the study focuses on the factual discourse on climate change that environmentally active youths contribute to and are a part of in environmental youth organizations’ social media channels. The discourse is understood as the context in and through which the social world is practically created, including actor categories, subject positions, and trust (Hajer, 1995). The study is guided by the following research questions: what subject positions are constructed by environmentally active youth in social media for the actor categories *science* and *youth*, how do subject positions within these actor categories relate to one another, and do these relations show trust in science?

The research questions are answered through an analysis of FFF in the movement’s origin country of Sweden, as well as of the environmental youth organization *Fältbiologerna* (literally translated as *the (Swedish) Field Biologists*), which is Sweden’s oldest and still largest nongovernmental organization that organizes youth to learn about, engage with, and advocate for nature (Jamison et al., 1990; Kaijser and Larsson Heidenblad, 2018; Larsson Heidenblad, 2021). These two organizations have been chosen because they are the largest and most active and influential organizations in Sweden through which youths themselves organize to defend the climate and the environment. Based on this choice of organizations, in this study, youths refer to the age group that is active in these organizations, that is, young people aged 6–26 (cf. Pickard, 2019).

The empirical material used for the analysis consists of FFF Sweden’s and Fältbiologerna’s Facebook posts during the 3-year period of 2019–2022. The posts are viewed as a collection of discursive extracts that together present the subject positions made available for science and youth in relation to science by the environmental youth organizations in their social media communications (cf. Bamberg, 2007; Hajer, 1995; Potter, 1996). The Facebook posts are understood as creating a snapshot of the discourses that FFF Sweden and Fältbiologerna take part in, contribute to, and make use of to construct social positions for science and themselves (cf. Salvador and Norton, 2011). These Facebook feeds thereby contribute to the reproduction and consolidation of a specific understanding of science–public relations and youths’ trust in science. Through this analysis, the study contributes knowledge of how one part of the discourse on climate change is constructed and of the subject positions made available for youths within it. This knowledge also becomes useful and significant as a reference and starting point when continuing the studies of the relation between science and environmentally active youths in other venues in which the discourse on climate change is also created, such as face-to-face interactions and other written material.

The article consists of six sections, of which this introduction is the first. In the second section, the study is contextualized by presenting FFF Sweden and Fältbiologerna, as well as situating the study with respect to previous research. The third section develops the paper’s theoretical framework on trust by describing the study’s key concepts: *actor category, subject position, category entitlement*, and *trust.* The fourth section presents the study’s method, research design, empirical material, and analytical strategy. Section five presents the analysis of the empirical material. The analysis identifies, explores, and explains the subject positions that are constructed by FFF Sweden and Fältbiologerna within the actor categories of *science* and *youth* in the discourse on climate change. The sixth and final section draws on the analysis to discuss and determine how these subject positions relate to one another in terms of trust. The concluding discussion shows how the relationship between *science* and *youth* is crucial for the construction of subject positions within these actor categories, as well as how these relations offer science credibility and make youths important actors in the discourse on climate change.

## 2. FFF, Fältbiologerna, and the larger context of the study

Following climate activist Greta Thunberg’s solo school strike for the climate outside the Swedish parliament in 2018, FFF emerged and grew as environmentally concerned youths in cities all over the world adopted this method to strike for the climate to raise awareness of the climate crisis and to protest current political climate actions (Olesen, 2022; Solli and Mäkitalo, 2022). The movement’s rapid growth as well as its global spread, visibility in media, and invited presence in political forums, have made the organization important in multiple respects. FFF has created a social space for youths who are worried about the climate crisis. FFF has allowed these youths to be a part of, engage with, and contribute to a local environmental community with strong global ties. FFF has put faces and voices to the otherwise invisible and abstract notion of the coming generations that will face the consequences of past and current societies’ decisions on how to use the Earth’s resources. The collective power of FFF has allowed youths to be recognized as stakeholders and participants in the public debate on climate change (Marquardt, 2020). Thus, by becoming organized, youths have created new discursive positions and social spaces, offering discursive room for new forms of identity development.

Fältbiologerna was founded in 1947 by and for youths between the ages of 6 and 26. The organization is driven by the objective of spreading knowledge about nature by enabling youths to learn about nature through hands-on activities, as well as through written material and self-organized classes. Thus, at the heart of Fältbiologerna, we find excursions as a method that aims to develop knowledge among the participants through seeing, hearing, and taking note of nature (Kaijser and Larsson Heidenblad, 2018). In addition to excursions in different forms, Fältbiologerna has, since the 1960s, also come to play an important role in the political debate on environmental issues as advocates for nature and its protection and preservation (Larsson Heidenblad, 2021). Fältbiologerna participates in activist actions in Sweden, as well as internationally, to protect biodiversity and counteract climate change. Fältbiologerna is a member organization that follows the traditional democratic structure of NGOs in Sweden. The organization relies heavily on voluntary work but has a few employees who work on national and sometimes district levels to support organizational development and communication (Klöfver, 1992; Larsson Heidenblad, 2021). In comparison, FFF Sweden is not a formalized NGO but a network of registered school strikes all over the country that relies exclusively on voluntary work.

Despite the long history of this organization, research on Fältbiologerna and its involvement in speaking for the climate is quite limited. In addition to studies on the participants of the organization that were published in Swedish in the 1990s (Klöfver, 1992, 1995), and studies of Fältbiologerna’s journal in the late 2010s and early 2020s (Kaijser and Larsson Heidenblad, 2018; Larsson Heidenblad, 2021), Fältbiologerna is only mentioned in previous research as one important organization among many in studies of the development of the modern environmental movement in Sweden in the 1960s and 1970s (Boström, 2001; Carle, 2000; Jamison et al., 1990). Looking at previous studies of FFF, the situation is completely different (Neas et al., 2022).

As FFF has grown internationally, so has research on this movement. Extensive literature can already be found on the development of FFF (e.g., Kern and Opitz, 2021; Olesen, 2022); the demographics and motivations of FFF participants (e.g., Cologna et al., 2021; Huttunen, 2021; Wallis and Loy, 2021); the framing of messages in speeches held at FFF school strikes and by major figures of the movement, primarily Greta Thunberg (e.g., Buzog’any and Scherhaufer, 2022; Marquardt, 2020; Molder et al., 2022; Sorce and Dumitrica, 2023; Svensson and Wahlström, 2023; Zulianello and Ceccobelli, 2020); media coverage of the FFF (e.g., Bergmann and Ossewaarde, 2020; Huttunen and Albrecht, 2021; Zabern and Tulloch, 2021); and FFF’s use of social media to raise awareness of climate change and mobilize participants to participate in school strikes for the climate (e.g., Boulianne et al., 2020; Haßler et al., 2023; Jung et al., 2020; Score, 2022; Soler-i-Martí et al., 2020). The different perspectives through which FFF has been studied show how the organization sits at the crossroads of multiple research fields that all have a long history of studying youth movements, such as sociology, political science, political sociology, youth studies, and social movement studies (e.g., Pickard, 2019). As previously mentioned, research on FFF’s and other environmental youth organizations’ relation to and understanding of science is still largely lacking, however. By aiming to address this lack of knowledge, this study will contribute to research on youths by adding to the more general discussion on the public’s trust in science.

The trustworthiness of science and its authoritative position in society has historically been a core interest of the philosophy of science and social science studies of science (Merton, 1973; Yearley, 2005). In the 1980s and 1990s, the notion of trust in science and the lack thereof became of specific importance for understanding science–public relations in the aftermath of environmental and technical disasters and problems in relation to which the trustworthiness of science was questioned, such as in the cases of the radioactive fallout from Chernobyl on the West Cumbrian hillsides (Wynne, 1989, 1996), the risks of bovine spongiform encephalopathy (also known as “mad cow disease”) (Irwin, 1995; Irwin and Wynne, 1996; Jasanoff, 2005), and the consequences of genetically modified foods (Irwin and Michael, 2003; Jasanoff, 2005). In recent decades, we can see similar instances of science’s trustworthiness being questioned regarding issues such as the extent to which human activities contribute to climate change (Fage-Butler et al., 2022; García Casañas, 2021), the safety of vaccinations (Larson et al., 2015), and most recently, the understanding and management of the COVID-19 pandemic (O’Doherty, 2023; Rughiniş and Flaherty, 2022). On all these issues, the public has negotiated their relation to science as part of the process of determining the trustworthiness of scientific knowledge and the role of both science and the public in managing these problems (cf. Eden, 2010; Eden et al., 2006). This study will contribute to this field by exploring and explaining how youths who are environmentally active in two different environmental youth organizations relate to science in social media, whether they trust science, and how youths’ relation to science creates a discursive context in which they develop their identity.

## 3. Theoretical framework

To explore how environmentally active youths relate to and trust science in social media, a theoretical framework will be used that allows the study to explore and explain the discursive construction of, as well as differences and relations between, actor categories and social positions (Michael, 1996). Thus, the key concepts in this framework are *actor category, subject position, category entitlement*, and *trust.*

The most general understanding of discourse as used in this study, is that it is a context in and through which the social world is constructed. It is through discursive constructions and negotiations that boundaries are drawn between social actor categories, knowledge systems, and whom to trust in telling the truth. This process of constructing actor categories ascribes credibility and category entitlement to some actors while undermining the credibility of others. In other words, “certain categories of actors are treated as entitled to know particular sorts of things, and their reports and descriptions may thus be given special credence” (Potter, 1996: 114).

Actors with category entitlement are collectively acknowledged as unique experts in their field, be they doctors in the field of medicine, carpenters in the practice of making furniture, or photographers in taking pictures. With this, it follows that having category entitlement makes someone entitled to speak with authority, not on their own merits, but because they are part of the actor category that has been identified and determined as entitled and capable of telling the truth. Thus, the person is trusted and acknowledged as an authority and given category entitlement because they have gone through the social gatekeeping processes that determine who is to be included in this specific actor category and who is not.

Throughout modern society, science has been acknowledged as a uniquely positioned actor category that is entitled and able to tell the truth (Beck, 1992). On the opposite side of science’s category entitlement, we find a general understanding of the public as a scientifically illiterate and ignorant actor category (Brossard and Lewenstein, 2010). Discursively, this public ignorance has been explained as being created through one of three different arguments (Michael, 1996). The difference between science and the public and the ignorance among the public is constructed and upheld first through the argument that ignorance among the public is the result of laypeople having a different mind than scientists, an argument that portrays the idea of an essential difference between scientists and members of the public. Second, the difference between these actor categories is upheld through the argument that it is not the public’s job to produce expert knowledge or manage expert systems, an argument that contributes to organizing society through a division of labor. Third, the difference is upheld through the argument that engaging with expert knowledge is not interesting or relevant to the layperson on a personal level and is thus best left for interested experts to take responsibility for. As a result of all three arguments, ignorance is incorporated into the public’s identity and forces the public to trust science, fortifying science’s category entitlement. Thus, science is identified as knowledgeable and actionable, while the public is identified as their dependents (Collins and Evans, 2007; Potter, 1996).

Following this construction of science as knowledgeable and entitled and the public as ignorant and reliant, the public in their everyday lives end up having to trust expert systems of various kinds, such as scientific knowledge systems and technical systems. Giddens specifically defined trust as “confidence in the reliability of a person or system, regarding a given set of outcomes or events, where that confidence expresses a faith in the probity or love of another, or in the correctness of abstract principles (technical knowledge)” (Giddens, 1990: 34). Following this definition, trust creates a hierarchical relation between science and the public in which science carries the power to tell the truth and the public must trust that the expert systems are working. In this way, trust becomes a form of boundary work that demarcates knowledge systems from one another, determines credible subject positions among these systems, and assigns category entitlement.

However, since the actor categories of science and the public as well as the character of the relation between them, are the result of discursive negotiations, these categories and their relation should be understood as always open to questioning and renegotiation. Science’s position as authoritative, able to tell the truth, and able to speak from a position of category entitlement can be and has been questioned, distrusted, and undermined, as previous research shows (see above). Thus, it cannot be taken for granted that these are the current relations that are being constructed by environmental youth organizations between environmentally active youth and science in social media. Instead, this is an empirical question that is yet to be answered using the concepts of *actor category, subject position, category entitlement*, and *trust* as analytical tools.

## 4. Research design and method

For its analysis, this study uses material collected from FFF Sweden’s and Fältbiologerna’s Facebook pages. The choice to focus the analysis on material published on Facebook and exclude other social media platforms has been made to allow an equal comparison between the two organizations’ communication on one of the largest social media platforms used in Sweden.^1^ The Facebook feeds include a wide variety of posts, including text (e.g., information on and reports from activities, as well as argumentative original texts and citations of texts published by the organization elsewhere), images (both illustrations and photos), and videos (e.g., live clips from activities and Zoom seminars). The study exclusively analyses the text and images. Thus, a choice was made to include the text posted with videos in the study but not the videos themselves.

The study includes posts made by the two organizations from November 2019 to October 2022. This period has been chosen to include half a year before and after the introduction and removal of Sweden’s restrictions concerning the COVID-19 pandemic, including material before, during, and after the pandemic restrictions were deemed important since the consequences of the restrictions have strongly affected the character of all climate youth activism, which was forced by the pandemic to move online, compared to being highly visible in the public space previously (cf. Haßler et al., 2023). Looking at the collected material, it is found that the character of the text, the actor categories articulated, and the relations between science and youth do not differ before, during, and after the pandemic. Instead, the discourse that is communicated is stable over the 3 years studied. The studied period also includes the Swedish parliament election in September 2022, including material before, during, and after the parliamentary election, which was deemed important since it was the first parliamentary election since 2018, when Greta Thunberg started her school strike and initiated the FFF movement. Thus, the election could be expected to intensify the organizations’ contributions to the public conversation on climate change. Looking at the collected material, the election is present in the Facebook feeds but does not qualitatively affect the character of the text, that is, the actor categories articulated or the relations between science and youth. The fact that both the pandemic and the election were noticeable in the material but that neither event affected the construction of the discourse was interpreted as support for the decision to use Facebook posts to obtain access to a piece of the general discourse on climate change and science–public relations in which the FFF Sweden and Fältbiologerna exist and to which they contribute.

All Facebook posts were published in Swedish and have been read in full by the author as part of a general analysis of the material. In a second step, the posts that referenced science were selected for further analysis of the discursive construction of actor categories, subject positions, category entitlement, and the relation between the positions in terms of trust. The posts selected for this in-depth discourse analysis were chosen on the basis that they directly or indirectly mentioned or referred to science, scientists, or scientific knowledge in any way. This could be by mentioning a specific scientist by name or title, accounting for or citing scientific results, or referring to or in any way arguing for “the importance of science.” Thus, the analysis is asymmetrical in that it studies all posts in which science is mentioned but not all posts in which the environmentally active youths come to show. The reason for this is the study’s interest in the youths’ relation to and trust in science. Thus, despite its general label—*youth*—the ambition of the study is not to give an exhaustive description of all subject positions within the actor category *youth.* Instead, the subject positions of interest for this study are the positions that the environmentally active youth in FFF Sweden and Fältbiologerna are creating for themselves as they relate to science.

In total, FFF Sweden made 229 posts during the studied period, of which 22 posts referred to science in any way and were included in the in-depth discourse analysis. In total, Fältbiologerna posted 1424 posts during the studied period, of which 118 posts referred to science in some way and were included in the in-depth discourse analysis. The analysis only included original posts made by FFF Sweden and Fältbiologerna, and no comments or reactions to these posts were collected or included in the analysis. This is because it is not dialog and interaction that the study is interested in but the organizations’ construction of the relationship between youth and science.

The empirical material selected for the discourse analysis was analyzed by the author in three steps. First, the actor category science was identified in the organizations’ discussion on climate change. Second, youth as an actor category was identified by analyzing how youths, in relation to science, were constructed as actors when discussing climate change. The analysis of both actor categories (science and youth) focused on how they were characterized and descriptively presented in the text. The characteristics included descriptions of what allowed someone to be part of an actor category and what agency, credibility, and category entitlement was given to different subject positions within these actor categories. Third, the actor categories were analyzed with respect to how they were positioned relative to one another to explore and explain their relation in terms of trust. The analysis explored and explained how environmentally active youths in Sweden relate to science on the topic of climate change and how this relation creates a discursive context in which youths develop their identity.

## 5. Results and analysis

### The actor category of science in the discourse on climate change

Starting the analysis with FFF Sweden’s Facebook feed, we can see how FFF Sweden primarily describes climate change as a crisis that is taken for granted. In comparison to Fältbiologerna, FFF Sweden seldom refers to individual scientists or explains or describes climate change in its posts. Instead, FFF Sweden’s posts are communicated in a way that takes the climate crisis as an unquestioned reality and assumes that the recipient of the post has a shared science-based understanding of climate change and the crisis it causes, or as an alternative, trust that scientists understand the crisis. This way of implicitly using science to situate the posts by taking its knowledge for granted contributes to creating and strengthening science’s *category entitlement*. Following on this, in instances when new research is reported on or referenced by FFF Sweden, this is also done in a way that requires the reader to have an awareness and basic scientific knowledge of how human activities are changing the climate to understand the news, or alternatively to trust that science holds this knowledge. These expectations of a shared science-based reality and *trust in science* are taken as a departure point when demanding that people in power should “listen to the best-unified science currently available” (FFF Sweden, 2023). This way of indirectly acknowledging and strengthening science’s category entitlement regarding climate change is exemplified by the following extract:

Today is the last day of 2020, what a year it has been—so different in so many ways. However, even though a lot has changed, the climate crisis is just as urgent as it was before the pandemic, and political inaction remains. The coronavirus pandemic has shown how people in power do have the power to act following science and to take crises seriously—there are no excuses anymore^2^ (FFF Sweden—December 31, 2020)

In the text extract, it is not recalled or described what science says about climate change; it does not even refer to climate science in any explicit way. Instead, the focus of the text is on “people in power” and their capacity to act on scientific knowledge in general. By so doing, without explicitly mentioning climate science, through the expressions “people in power do have the power to act following science” and “there is no excuse anymore,” FFF Sweden constructs, confirms, and cements climate science as holding a superior *subject position* and creates a *linear relation* through one-way communication with society and people in power, who are positioned as receivers of the message who are supposed to listen to the knowledge that science is offering.

This strategy of confirming and strengthening science’s credibility by taking its knowledge contribution for granted as the normal understanding of reality can also be seen in Fältbiologerna’s Facebook feed. However, explicitly referring to research results or individual scientists and making use of scientific knowledge has a much larger presence in Fältbiologerna’s posts. Scientists are explicitly referenced as holding a *subject position* with *category entitlement*, either individually or collectively. These references are made to strengthen the credibility of specific knowledge claims and arguments made in the posts about the effects of changes to the climate or to give attention to newly published research results. This way of positioning science sporadically also appears in FFF Sweden’s Facebook feed.

In addition to this explicit referencing of science, individual scientists and their knowledge have a strong presence in Fältbiologerna’s feed through posts that advertise and report on digital lectures, especially during the COVID-19 pandemic. During certain periods of the pandemic, Fältbiologerna organized weekly lectures with guests, among whom many were researchers or highly educated professionals. The following extract concerns the first presentation of this lecture series:

The threats of climate change and the extinction of species do not stop during the Coronavirus crisis. Our fight for the climate and biodiversity must continue—but take a new form, in solidarity with at-risk groups. Hence, Fältbiologerna is, during the crisis, now making a big new launch in public education: “An evening of activism.”

“An evening of activism” is a series of digital lectures—open to everyone, of all ages—about different environmental questions and environmental activities. Each Thursday, a new activist, researcher, or debater will give an educational and engaging public lecture on a topic, ranging from systemic critique and mines to climate denialism and questions about the ocean. (Fältbiologerna 29 April, 2020)

This text extract starts similarly to the one from FFF Sweden above. Both posts point to the ongoing nature of the climate crisis and argue for the need to continue fighting it. However, whereas FFF Sweden uses science to contextualize its focus on inaction among people in power, Fältbiologerna to a larger extent engages with the scientists and the knowledge they produce to allow youths (and others) to enhance their knowledge on the topic. Through lectures such as these and posts about other activities such as excursions and species inventories, Fältbiologerna emphasizes the content of the scientific results, the process behind them, and how they can be understood and used by others. Thus, by explicitly referencing scientists, Fältbiologerna uses a second discursive strategy to create a *subject position* within the actor category of science. The subject position is that of a teacher who provides important and empowering knowledge to youths (and others) and confirms science’s prominent position in the discourse on climate change.

In both FFF Sweden’s and Fältbiologerna’s Facebook feeds, science holds a prominent position with a strong *category entitlement*. The knowledge produced in and communicated from the position of science is acknowledged as the truth that describes the world. This position is created and confirmed in one of two ways: first, by situating the Facebook posts in a context in which science’s description of climate change as a crisis is taken for granted, sometimes without even explicitly mentioning science; and second, by citing scientific knowledge or explicitly referring to research results and to scientists as a collective or individuals. Thus, taken together, FFF Sweden and Fältbiologerna both contribute to the creation of *two different subject positions within the actor category science*: (i) the starting point when advocating for action and (ii) a source of learning and empowerment. These two positions contribute to strengthening both the credibility and category entitlement of science. By ascribing both of science’s subject positions a unique capacity to create truthful knowledge, science is discursively made the key actor category in the discourse on climate change.

### The actor category of youth in the discourse on climate change

The discourse analysis shows that the *two subject positions* within the actor category of science are mirrored by *two subject positions* within the actor category of youth. First, the position of science and scientific knowledge as *the starting point when advocating for action* creates an *activist and science champion subject position* for youths. Second, the position of science and scientific knowledge as *a source for learning and empowerment* creates a subject position for youths of *being knowledge holders and knowledge producers*. Since both subject positions for science are found in both FFF Sweden’s and Fältbiologerna’s Facebook feeds, so are the two subject positions for youths. However, while the subject position of being an activist and science champion heavily dominates FFF Sweden’s posts, Fältbiologerna’s posts involve a mix of the two in which the position of being a knowledge holder and knowledge producer (knowledge holders/producers) is both a standalone position and a steppingstone toward becoming an activist.

*The activist and science champion* is the key *subject position* for youths in FFF Sweden’s posts. In this position, the youth’s mission and responsibility is to push leading politicians to take science-based action, which contributes to upholding a *linear relation* of communication between science and society in which science speaks truth to power (cf. Zulianello and Ceccobelli, 2020). This mission is described as a fight for justice that is primarily fought on the streets but that during the pandemic had to move online. The *subject position* and the collective notion of how the position is enacted are shown in the following extract:

Today, it is both Valentine’s Day and one year since the last national climate strike before the COVID-19 pandemic came to Sweden. That day, we were many thousands on the streets of Sweden to demand climate justice from our political leaders, something we have not been able to do since. But it is more important than ever that we continue the fight. It is more important than ever to put pressure on politicians to counteract the climate crisis. [. . .] We are hoping that we will be able to gather on the streets again soon to strike, but in the meantime, we need to treat all crisis with the seriousness it deserves and listen to the science and the experts. (FFF Sweden, 14 February, 2021)

As in many of FFF Sweden’s other Facebook posts, this text was posted with pictures of youths on strike. In this specific post, the accompanying pictures are from the strikes in Stockholm, Gothenburg, and Sundsvall on February 14, 2020. The pictures reinforce *the collective notion of the activist and science champion subject position*, showing people who are gathering and marching on city streets carrying signs that address the severity of the climate crisis and demand action. This way of visualizing the subject position of being an *activist and science champion* is a recurring one in FFF Sweden’s Facebook feed: youths with protest signs, pictured together in groups (before and after the pandemic) or on their own (during the pandemic and often posted by FFF Sweden with #VeckansSkylt (#TheSignOfTheWeek) or grouped in collages). In these pictures, the protest sign is what *discursively makes* the youth an *activist and science champion*. The repetition established during the pandemic of the picture format of photographing oneself holding a protest sign offers a template for youths of what is required to take the subject position of an activist. *To pass* as an *activist and science champion*, someone needs to express their demand for climate justice and political action on a sign and join the collective movement on the streets or social media (for example, by posting their picture on Twitter under #ClimateStrike).

Fältbiologerna also encourages its members and readers to participate as activists in FFF Sweden’s strikes but does not organize any strikes itself. Instead, its posts show *two additional ways of being an activist and science champion* for the climate. The first way is by participating in environmental debates and discussions. As a member of Fältbiologerna, this could be done either as a participant in one of its events or by representing the organization as a guest speaker and debater in external events, ranging from panel discussions at conferences to political hearings. The second way is by participating in protests that target climate-harming practices and organizations, for example, blocking entrances to facilities related to oil production and logging areas. These protests are often organized in collaboration with other environmental organizations.

However, the *main subject position* offered to youths within Fältbiologerna is to be a knowledge holder/producer. The main focus of the Facebook feed is to invite and make room for youths to learn about nature and the climate and for them to contribute their knowledge to help others learn. Traces of this subject position were seen in the analysis above of the actor category of science, such as reports from and engagement in scientific methods for knowledge production through, for example, excursions and species inventories. This position is prevalent in Fältbiologerna’s Facebook feed but also sporadically appears in FFF Sweden’s feed. This subject position is made more visible in the following extract:

Education for the people!

The objective of public education is not only to quench a thirst for knowledge but also to provide citizens with knowledge in a democratic and egalitarian manner. With the help of knowledge, we can develop, widen our perspectives, and create change!

Public education is found at the core of Fältbiologerna and depends on the engagement of individual members. For some time now, the digital library “*the Public Education Library”* has existed, filled to the brim with resources for those of you who want to learn more about everything, from the climate—to knowledge of species—to how you manage your own section within Fältbiologerna. Everything is available on our website. *The Public Education Library* is open for all members—and those of you who want to are more than welcome to contribute your knowledge! (Fältbiologerna, 9 February, 2022)

The *subject position* of being a *knowledge holder/producer* is created in relation to the positioning of science as a practice that contributes important and trustworthy knowledge that is open for everyone to learn and the positioning of scientists as people to learn from. The relation between these positionings of science and youth is a two-way relation. Despite the difference in the formal credentials that are required to be a part of the actor category of science, the subject position of being a *knowledge holder/producer* is open to and shares science responsibility to produce knowledge following the logic and rules of science. Thus, even though scientists are acknowledged as having more knowledge and experience, both scientists and youths are seen as contributing to the same objective: to create a better understanding of the environment and the problems it faces in the context of climate change. Instead of having a *linear relation* to science, as in the case when youths are positioned as *activist* and are supposed to listen to science as *the starting point* for their advocacy and *science championing*, the *subject position* of youth as a *knowledge holder/producer* are situated to science as *a source for learning* in a shared *space of knowledge production.* What determines the relation between the scientists and youths in this mutual space of knowledge production is the amount of experience and volume of knowledge rather than a difference in assigned tasks or any essential differences in disposition or ability to create truthful knowledge. Instead, the *subject position* of being a *knowledge holder/producer* is open for youths with any level of climate change knowledge, from those with no experience or knowledge in the field to well-read university students whose body of knowledge borders that required to be included in the actor category of science.

In Fältbiologerna’s Facebook feed, combining being an *activist and science champion* with being a *knowledge holder/producer* is encouraged. Combining learning from and about nature with fighting for nature is central, as Fältbiologerna’s activities are described collectively, such as in the following extract:

We are outside in nature and inside the environmental debate—this is both because nature gives us energy and inspiration to keep fighting and also because experiencing the flight of the meadow hawk, the smell of the diamond willow fungus, and the wolf howl has its value. We stand for nature’s rights. (Fältbiologerna, 5 January, 2022)

The possibility of *moving between* these positions of *learning from and producing knowledge* of nature and being an *activist and science champion* in environmental debates allows youths to gain a stronger position, legitimacy, and empowerment in the position of an activist. Moving between the two subject positions and developing their own knowledge allows youth to step into the role of an activist and science champion not only based on a *trust in the expert system of science* but also encouraged by *trust in their own capacity* to make scientifically based knowledge claims about what needs to be done concerning the climate.

Taken together, FFF Sweden and Fältbiologerna contribute to the creation of *two different subject positions for youths*: (i) being an activist and science champion as well as (ii) being a knowledge holder/producer. These two positions are both created in relation to science, where the activist is primarily situated as a science champion with a *linear relation* to science, whereas the knowledge holder/producer is situated in the same *space of knowledge production* as a scientist, with a two-way relation to the actor category of science. Both positions offer a collective type of belonging for youths in which they are allowed to be active and contribute, either through advocacy or knowledge production.

## 6. Discussion and conclusion

By studying the part of the discourse on climate change that is constructed and becomes visible in environmental youth organizations’ social media channels, this study has made it evident that the character of the relationship between the two actor categories *science* and *youth* is crucial for the construction of the individual subject positions within these categories in the discourse on climate change. The character of the relationship between science and youth creates and reveals spaces where trustworthy knowledge on climate change may be produced and sets boundaries that determine what subject positions are to be trusted on the topic and by whom. The character of the relation between science and youth creates subject positions open for youths to act from and to discursively develop their identity within.

As the analysis has shown, the dominant, but not exclusive, subject positions within FFF Sweden’s Facebook feed are that science is constructed as the position from which a taken-for-granted truth is told of how to understand and manage climate change, and youths are positioned as activists and science champions who want to impact the discourse on climate change and the management of the climate crisis. Thus, there is no tension between science and activism. Instead, scientific knowledge is a prerequisite to activism. The two positions are created through a linear relation upheld by the youth’s trust that science has a unique capacity to produce truthful knowledge that gives science a specific responsibility and position to produce expert knowledge that is to be used in the management of climate change. The different capabilities and responsibilities attached to the two subject positions through this form of trust provide a background and rationale for the youths’ demand for politicians and the adult generation to “listen to the science.” This demand becomes a shorthand description of the division of labor in which science is trusted to provide truthful and relevant knowledge for policy-makers and the rest of society to act upon. Importantly, this division of labor does not lead to the passivation of youth. Locating the responsibility to create knowledge and truth within science does not mean that youths are left without responsibility. Instead, the linear relation between science and the activist as science champions, as well as science and policy, makes the youth activist responsible for *listening* to science, *embracing* its message, *acting* on it, and *making other people aware* of it. Thus, this form of trust in science is not passive or powerless. Instead, it generates an active and important role for the activist as one who holds the responsibility to engage in and encourage action.

The dominant, but not exclusive, subject positions in Fältbiologerna’s Facebook feed are that science is positioned as a source for understanding climate change and youths are positioned as knowledge holders and knowledge producers, a position that shares characteristics with the scientific position in that it follows scientific principles regarding how to produce knowledge as well as empowering functions. Science as a source for learning and youths as knowledge holders/producers are created through a relation in which they share the same objective, collaborate, and engage in the same practice to produce and communicate truthful scientific knowledge that is to be used in the management of climate change. Thus, this collaborative relation acknowledges the scientific practice of producing knowledge as uniquely capable of producing truthful and trustworthy knowledge. However, in contrast to the linear relation to science, which creates a division of labor in which only scientists are responsible for the practice of scientific knowledge production, the collaborative relation between science and youths blurs the boundary between the actor categories in that it makes both scientists and youths responsible for the practice of scientific knowledge production. The understanding that everyone, regardless of current knowledge on the topic of climate change, can engage, at least to some extent, in scientific practices and knowledge production opens the possibility for youths (and others) to not only *listen* to science but also *learn* from, *understand*, and *contribute* to science. Trust is not placed in science according to an argument that scientists have a different mind than laypeople or that science has a specific task to perform (cf. Michael, 1996); instead, trust is placed in science according to an argument that the character of the scientific practice itself results in trustworthy knowledge (cf. Merton, 1942).

Furthermore, the analysis has shown how environmental youth organizations, in relation to their notion of science, have created discursive positions and social spaces, offering discursive room for the youths to engage in identity development. Both subject positions available for youths in relation to climate science—*activist and science champion* as well as *knowledge holder/producer*—create discursive and social spaces for youths to be active subjects who develop their identity. As activists and science champions, youth develop their identity as political actors with an acknowledged role in the climate debate. As knowledge holders/producers, youth take on the same characteristics ascribed to scientists as credible knowledge producers. The analysis also shows how youths may cultivate both identities of being an *activist and science champion* as well as *knowledge holder/producer* and how they move between the subject positions. For example, the knowledge created by a knowledge holder/producer can be used to gain a stronger footing and legitimacy when stepping into the position of being an activist and science champion. By identifying with the characteristics ascribed to science’s position as *a source for learning*, youth also strengthen their alignment with science as *the starting point* when advocating for action as *science champions*. As they move between the two subject positions—*activist and science champion* and *knowledge holder/producer*—youths are constructed as people who, alongside scientists, are to be trusted by other actor categories within the public and the policy arena on the topic of climate change. Youths do not become scientists, but the subject positions of *knowledge holder/producer’s* alignment with the characteristics of scientists as *a source for learning* makes it possible for youths as *activists and science champions* to act with category entitlement from a similar position as scientists to speak the truth about climate change.

In sum, by adopting a discourse analytical perspective to explore and explain youths’ relation to climate change science in FFF Sweden and Fältbiologerna, this study has shown how environmentally active youths trust science in ways that offer science both credibility and a strong category entitlement. The study has further shown how this trustful relation at the same time makes the youths themselves important actors in the discourse on climate change as both capable of producing knowledge on the climate crisis and advocating for science-based action. With these results, the study contributes to filling gaps in previous knowledge on both environmentally active youth and the public’s trust in science. The study contributes knowledge on youth as a previously understudied segment of the public in the field of social studies of science–public relations (cf. Eden, 2010; Eden et al., 2006) and complements our understanding of youths’ motives to engage in environment and climate issues through revealing how the youths’ trust in science also creates possibilities for their one identity development (e.g., Cologna et al., 2021; Huttunen, 2021; Wallis and Loy, 2021). Finally, the study expands our knowledge of environmentally active youths’ relation to other actor categories than the previously most noticed and studied; *political representatives* and *the adult generation* (cf., e.g., Whalström et al., 2020; Solli and Mäkitalo, 2022). Thus, the study reveals the larger complexity and new dimensions of these youths’ role, position in, and contribution to the discourse on climate change.

## Notes

1. FFF Sweden has social media accounts on Facebook (12k followers), Instagram (23k), YouTube (fewer than 100), and Twitter (4k). Fältbiologerna has accounts on Facebook (14k) and Instagram (8k). In the early stages of the study, a third organization that only uses Facebook was considered, making Facebook the only available choice for an equal comparison. However, as the study progressed, the amount of material from the third organization turned out to be too small to be analytically and ethically sound to include in the final analysis. Thus, the decision was made to focus the study on FFF Sweden and Fältbiologerna exclusively. Even though they also have Instagram in common, the frequent use of Facebook and the overlapping posts on the two platforms still make the initial choice to focus on Facebook valid.

2. All text extracts used as examples in this analysis have been translated from Swedish to English by the author.
